# VSGdb: a database for trypanosome variant surface glycoproteins, a large and diverse family of coiled coil proteins

**DOI:** 10.1186/1471-2105-8-143

**Published:** 2007-05-02

**Authors:** Lucio Marcello, Suraj Menon, Pauline Ward, Jonathan M Wilkes, Nicola G Jones, Mark Carrington, J David Barry

**Affiliations:** 1 Wellcome Centre for Molecular Parasitology, University of Glasgow, Glasgow Biomedical Research Centre, 120 University Place, Glasgow G12 8TA, UK; 2 Department of Pathology, Henry Wellcome Building, School of Medicine, Cardiff University, Heath Park, Cardiff, CF14 4XN, UK; 3 Department of Biochemistry, University of Cambridge, 80 Tennis Court Road, Cambridge CB2 1GA, UK

## Abstract

**Background:**

Trypanosomes are coated with a variant surface glycoprotein (VSG) that is so densely packed that it physically protects underlying proteins from effectors of the host immune system. Periodically cells expressing a distinct VSG arise in a population and thereby evade immunity. The main structural feature of VSGs are two long α-helices that form a coiled coil, and sets of relatively unstructured loops that are distal to the plasma membrane and contain most or all of the protective epitopes. The primary structure of different VSGs is highly variable, typically displaying only ~20% identity with each other. The genome has nearly 2000 *VSG *genes, which are located in subtelomeres. Only one VSG gene is expressed at a time, and switching between *VSG*s primarily involves gene conversion events. The archive of silent *VSG*s undergoes diversifying evolution rapidly, also involving gene conversion. The VSG family is a paradigm for α helical coiled coil structures, epitope variation and GPI-anchor signals. At the DNA level, the genes are a paradigm for diversifying evolutionary processes and for the role of subtelomeres and recombination mechanisms in generation of diversity in multigene families. To enable ready availability of *VSG *sequences for addressing these general questions, and trypanosome-specific questions, we have created VSGdb, a database of all known sequences.

**Description:**

VSGdb contains fully annotated *VSG *sequences from the genome sequencing project, with which it shares all identifiers and annotation, and other available sequences. The database can be queried in various ways. Sequence retrieval, in FASTA format, can deliver protein or nucleotide sequence filtered by chromosomes or contigs, gene type (functional, pseudogene, etc.), domain and domain sequence family. Retrieved sequences can be stored as a temporary database for BLAST querying, reports from which include hyperlinks to the genome project database (GeneDB) CDS Info and to individual VSGdb pages for each VSG, containing annotation and sequence data. Queries (text search) with specific annotation terms yield a list of relevant VSGs, displayed as identifiers leading again to individual VSG web pages.

**Conclusion:**

VSGdb  is a freely available, web-based platform enabling easy retrieval, via various filters, of sets of VSGs that will enable detailed analysis of a number of general and trypanosome-specific questions, regarding protein structure potential, epitope variability, sequence evolution and recombination events.

## Background

The variant surface glycoprotein (VSG) is essential for the survival of *Trypanosoma brucei *in mammalian hosts. There are ~5.5 × 10^6 ^VSG homodimers per cell and the cell surface monolayer that the VSG forms is considered to provide general protection from innate immune mechanisms [1,2]. The coat nevertheless elicits a specific, trypanocidal immune response. This is countered by antigenic variation, in which trypanosomes switch to expression of a distinct VSG which, if antigenically novel, allows clonal proliferation of the switched cells, generating a new parasitaemia peak. Each trypanosome expresses only one VSG gene but has the potential to switch to any of probably hundreds of others [3,4].

The VSG is a structural paradigm for α helical coiled coil proteins and for B cell epitope variation [5,6]. This is because its hundreds or thousands of isoforms have limited similarity in peptide sequence and antigenicity, but strong conservation in higher level structure. In *T. brucei*, mature VSGs contain 400 – 500 amino acids, most having between 420 and 460 residues. Most of the protein is an N-terminal domain of ~350 residues, which is followed by a C-terminal domain, containing one or two smaller subdomains of 40–80 residues each [5,7]. N-terminal domains usually have only ~20% identity between different VSGs, although some are more closely related. The most conserved primary structure feature is the cysteine pattern, of which there are three, resulting in the classification of this domain into types A, B and C [5]. In contrast to the extensive sequence diversity, secondary structure potential is conserved, with a consequent overall similarity between the N-terminal domains of distinct VSGs. The backbone comprises two long, antiparallel α helices that form a coiled coil. It is not known exactly which elements of this domain contain the exposed epitopes that are the basis of antigenic variation, but it has been inferred that they are conformational rather than linear and are located in the loops exposed at the most extracellular end of the N-terminal domain, outwith the helices [8-10]. One important question that is yet to be definitively answered is why N-terminal domains vary over their entire sequence, rather than in just the region encoding the exposed protective epitopes.

The C-terminal domain is hidden from antibodies (NJ & MC, unpublished), presumably due to its membrane-proximal location, so is thought not to contribute to antigenic variation. It has ~40% identity between different VSGs. The genome project has revealed that, rather than the four C-terminal domain types (1 – 4) previously recognized, based on their cysteine patterns, there are six types. Types 2, 4 and 5 are single domains, each containing four cysteine residues, whereas types 1, 3 and 6 contain eight cysteines and appear to be composed of two subdomains, each containing four cysteines. Individual VSGs can have any combination of N- and C-terminal domains (A1, A2, A3, A4, B1, B2 etc.), and, as judged from the VSGs analysed so far, there appears to be no restriction on combinations. At its C-terminal end, this domain contains a signal sequence for the addition of a glycosylphosphatidylinositol- (GPI-) anchor [2]. Although sequence features specifying GPI signal sequences have been identified [11], their full diversity across VSGs is not known, and study of as many as possible potential signal sequences could enable deeper understanding.

Despite about 1000 VSG sequences being currently available, mainly through genome and cDNA sequencing, it is not facile to retrieve a complete set from general databases. We have therefore created a database allowing retrieval of criterion-based subsets. This should facilitate a more detailed analysis of VSG structure and more general questions about protein structure including:

- what are the sequence requirements for coiled coil structures?

- can epitope diversity be correlated with diversity in primary and higher-order structure?

- How diverse are GPI-anchor signal sequences?

- How does evolutionary selection for diversification fit within a conserved protein structure?

It is worth noting that relatively few of the silent *VSG*s sequenced in the genome project are considered to be fully functional, and verification of function of any element, for example GPI anchor signal sequences, requires demonstration of expression. In contrast, the non-genome *VSG *sequences are based mainly on expression, most having been derived from cDNA sequences, and query returns in FASTA format report their derivation from cDNA or genomic DNA.

At the genetic level, trypanosomes use a strategy common to antigenic variation in a diverse range of microbial pathogens: accessing an archive of silent genes. In *T. brucei*, there is a large archive of silent, distinct *VSG *genes, effectively all of which are telomeric and subtelomeric. In the genome strain, about 1600 *VSG*s are arranged as tandem arrays in subtelomeres of a range of chromosomes, and it is likely that different strains contain substantially larger archives [12]. Only 4.5% of this set are annotated as intact genes, the rest consisting of atypical genes (do not convincingly encode maturable VSGs), pseudogenes (include frameshifts and/or stop codons), and *VSG *fragments. Another set, of up to ~200 genes, are located telomere-proximally in the ~100 minichromosomes; so far, based on three genes [13,14], this set appear to be intact *VSG*s. Despite the enormous size of the silent archive, each trypanosome expresses only one VSG. Expression occurs only from specialized, telomere-proximal transcription units termed bloodstream expression sites (BESs). For archival genes, activation therefore involves duplication into a BES at the expense of the previously transcribed *VSG*, which is lost. Even pseudogenes can be activated this way, as part of their sequence can contribute to the formation of mosaic genes. Although it is known that homologous recombination participates actively in *VSG *switching, it is thought that limited sequence homology within the coding sequence is involved in the formation of mosaic genes, and an important function of a database could be enabling identification of such sequence homologies [15]. Duplication of intact *VSG*s apparently can utilize homologous, imperfect repeats upstream of most *VSG*s and can end at the other flank in conserved sequences towards the 3' end of the coding region or further downstream, where the 3' untranslated region is encoded. Sometimes the incoming gene duplicate inherits part of the C-terminal domain encoding sequence from the *VSG *already in the BES.

The *VSG *archive is very diverse, to the extent that different trypanosome strains have widely different gene sets. How the archive evolves is unknown, but it is evident from the dispersed nature of the *VSG *gene arrays [4], and from analysis of duplication events within the archive (LM, JDB, unpublished), that homologous recombination, involving primarily gene conversion, plays a major role. It is now becoming clear that subtelomeres of various organisms, including humans [16], are preferential sites for the rapid evolution of multigene families [17], possibly due to the preferred use of particular recombination mechanisms [18]. Due to the availability of the sequence of most *VSG*s in the silent gene arrays, the trypanosome has now become an experimentally tractable paradigm for the role of subtelomere recombination in multigene family diversification. Thus, ready accessibility to the individual gene sequences in a dedicated database can help address a number of questions about chromosomes and recombination, such as:

- How do sequences spread and diversify within and between subtelomeres?

- What is the contribution of partial gene conversion, (micro)homologous recombination and point mutation to diversification of the gene family?

- What is the rate of evolution of coding sequences and of pseudogenes that can donate partial coding information?

## Construction and content

VSGdb has been constructed as a specialised database to store a definitive, annotated set of VSG sequences that can be retrieved for analysis at the nucleic acid or peptide level. The source sequence data include the genome sequence and other cDNA and genomic sequences in public databases. For the genome project sequences, final annotation was achieved through Artemis [19] and VSGdb shares all identifiers and annotation with the genome project database at geneDB [20]. Of necessity, due to the limits of empirical knowledge, annotation in the genome project is parsimonious, with features being scored as negative if there is any doubt. This applies in particular to GPI anchor signals, where manual annotation based on known VSG sequences was undertaken, allied with the parameters of the bigPI [21] and DGPI [22] prediction programmes. The GPI signals appear to be much more varied amongst the silent array genes than in the expressed set of genes available to date, so a conservative annotation approach was taken, envisaging stringent requirements for expression. As biochemical knowledge improves, it will be possible to evaluate more directly the stringency of this crucial surface anchoring process, and indeed VSGdb should be a catalyst for such biochemical study. A parsimonious approach has been taken also for the potential structure of the C- terminal domain. It has been assumed that a full set of four or eight cysteines would be a requirement for a correctly folded C-terminal domain to contribute to an expressed VSG: there are only few examples of C-terminal domains completely devoid of cysteines, and as yet no instance of domains with one to three cysteines has been recorded. Therefore, intact domains lacking these conserved features have been described as "atypical", in a grey area that is intermediate between putative functional and pseudogene domains.

**Figure 1:**
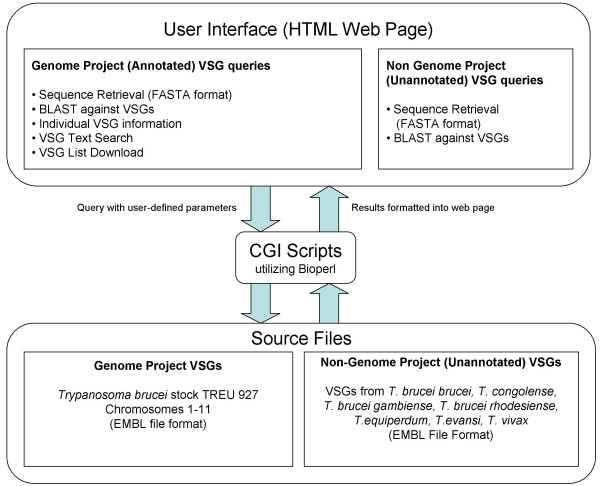
Information flow in a VSGdb query. The user inputs a query with defined parameters through a web interface, which is processed by CGI scripts to extract information from the source files and present the results as a web page.

**Figure 2:**
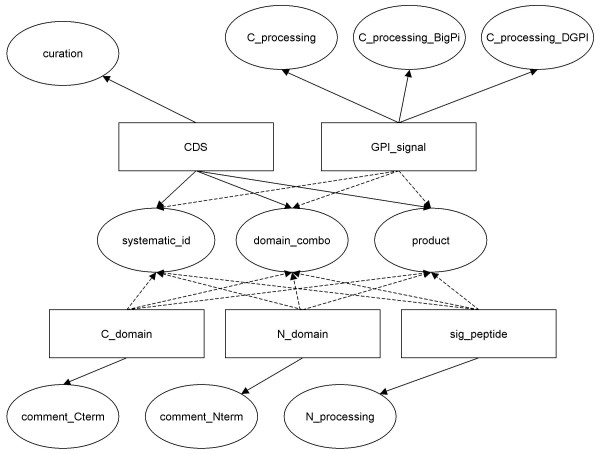
Annotation components utilized by VSGdb. The rectangles represent the primary tags which define parts of a VSG (CDS defines an entire VSG). The ovals represent features of each primary tag. Secondary annotation was carried out to link the systematic_ ID, domain_combo and product features of each VSG to all of its components. These features are used in the sequence retrieval (as FASTA or as a database for BLAST). All features are used in the construction of individual VSG pages. Features containing curation and comments are used in the text search.

VSGdb is freely accessible [23]. The user interface consists of web pages allowing users to query the database in several different ways. Queries are handled by CGI scripts that extract information from the source files and return them to the user in dynamically created web pages. Figure 1 shows the flow of information to and from the database. The source files are essentially of two types. The first type constitutes the majority of files currently present in the database and are EMBL-format sequence files [24] of chromosomes 1–11 and contigs of *Trypanosoma brucei *stock TREU 927, the genome of which has been sequenced [20]. Primary annotation of VSGs was carried out via the Artemis annotation software, and secondary annotation of the sequence files was then carried out manually to enable extraction of sequences of different parts of the VSGs and to make the source files parsable by BioPerl [25] modules. Details of the annotation can be seen in Figure 2. The second type of source files comprises EMBL-format sequence files of VSG cDNAs and genomic DNA sequences, some of which have been annotated, independently, from various trypanosomal species. These files were obtained from public databases.

The CGI scripts that process queries and return results were written in Perl and utilize BioPerl modules that parse EMBL-format files, and return sequence information quickly and accurately. It is due to the availability of these modules that it was decided initially to run the scripts on sequence files, but it is planned to move across to a standard database management system like MySQL. We shall update the database annually.

## Utility and discussion

**Figure 3:**
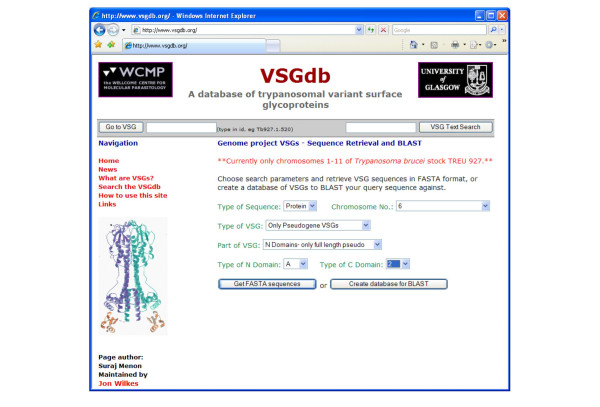
Screenshot of the web form to define parameters for retrieval of sequences from genome project (annotated) VSGs in FASTA format or as a temporary database against which to BLAST a query sequence. For non-genome project (unannotated) VSGs, only the type of sequence and the organism need to be specified.

The following features are available in the VSGdb for both the annotated genome project VSGs and the non-genome project VSGs:

• **VSG sequence retrieval **– The user is allowed to choose from several parameters and retrieve sequence data from the source files in the popular FASTA format [26]. For the annotated VSGs, these parameters include the type of sequence (DNA or protein), one or all chromosomes or contigs, the type of VSG (functional, pseudogene, etc.), the part of the VSG (full-length VSG, N-terminal domain, etc.), and the types of N- and C-terminal domains. Selection for N-terminal domains allows retrieval of the whole set of putative α-helical coiled coil sequences, or subsets thereof. For the VSGs not from the genome project, only the full sequence is returned. The user can select sequences from this set on the basis of species and strain/VSG repertoire, and can choose linkage to other databases. Figure 3 shows a screenshot of the web form where the user can select parameters.

• **VSG BLAST **– Using the same list of parameters as above, the user has the option of using the sequences retrieved as a temporary database against which to BLAST [27] their own query sequence. For genome project VSGs, the BLAST reports also include hyperlinks to GeneDB CDS Info [20,28], and VSGdb pages for individual VSGs.

**Figure 4:**
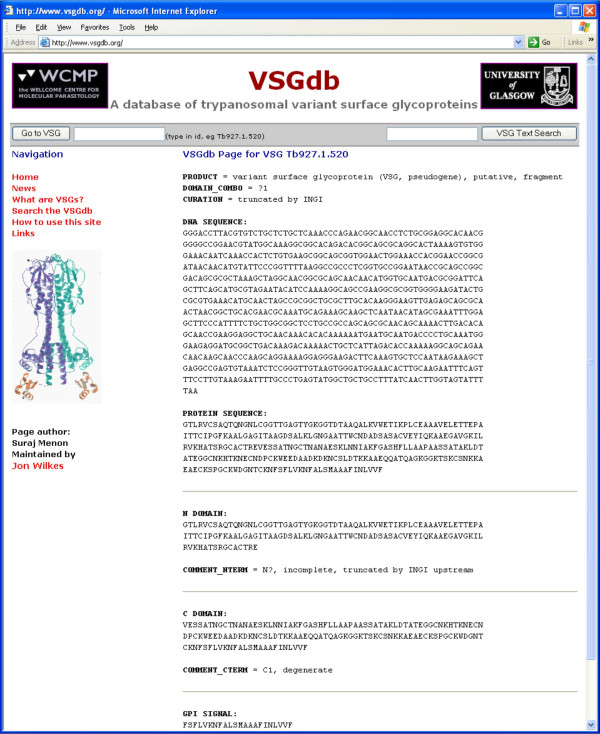
Screenshot of an individual VSG page (here Tb927.1.520). It contains all the sequence and annotation information available within the VSGdb for this particular VSG. Individual VSG pages can be accessed by typing in the VSG identifier in the searchbar or through the results of the text search and VSG BLAST functions. These are available only for annotated VSGs.

VSGdb also has the following features:

• **VSG Text Search **– This utility searches through annotation terms containing comments, curation and other information for search terms input by the user. Currently it treats multiple terms as one, analogous to putting quotes around search terms in a Google search. It outputs a list of identifiers of VSGs where there are hits, each of which is a hyperlink to the individual VSG web page.

• **VSG List Download **– This allows a user to input a list of VSG identifiers and retrieve various types of data regarding those VSGs. This is available only for genome project VSGs, since they are annotated.

• **Individual VSG Web Pages **– These contain all the annotation and sequence data regarding any one VSG and are accessible either by user-input of the VSG identifier or through result pages of the VSG BLAST and text search utilities described above. Again, these are available only for genome project VSGs, since they are annotated. The pages also have links to the corresponding GeneDB CDS info pages. Figure 4 shows a screenshot of an individual VSG page.

It is our aim that this facility will be of general and specific use. Its quality and development depend on feedback from users, and all contributions and suggestions will be most welcome.

## Conclusion

VSGs and their genes are important for trypanosome survival and growth, but also display features of general biological interest. Because the family has expanded and diversified very extensively, it is a unique biological resource for addressing questions about protein structure, evolution and genetic mechanisms. VSGdb allows all VSG sequence and annotation data to be accessed via a user-friendly, web-based interface. The database can be queried using various criteria, and retrieval, at either protein or nucleotide level, includes specific information on each VSG, especially the large set fully annotated in the genome project. Retrieval of subsets as temporary databases allows further detailed analyses. Besides contributing to general areas of biology, VSGdb should help enhance our understanding of trypanosome biology.

## Availability and requirements

Project name: VSGdb: a database of trypanosomal variant surface glycoproteins

Project home page: 

Operating systems: HTML 4.x-compliant browsers

Programming language: server side processing via perl; server Apache 2.0.40

Licence: none

## Authors' contributions

LM provided community-end annotation of all genome project VSG sequences and interacted with programmers to develop the database and user interface. SM, PW and JMW programmed the database and interface and installed the sequence files. NGJ and MC provided specialist knowledge on VSG sequences. JDB oversaw the project and helped define user requirements. All authors read and approved the final manuscript.
